# IMPACT OF RESPITE SERVICES ON DEMENTIA CAREGIVERS’ NEUROCOGNITIVE AND PREDEATH GRIEF OUTCOMES: A PILOT STUDY

**DOI:** 10.1093/geroni/igae098.2131

**Published:** 2024-12-31

**Authors:** Sydnie Schneider, Lauren Elliott, Elisabeth McLean, Volker Neugebauer, Keegan Diehl, Jonathan Singer

**Affiliations:** Texas Tech University, Lubbock, Texas, United States; Texas Tech University, Lubbock, Texas, United States; Texas Tech University, Lubbock, Texas, United States; Texas Tech University Health Sciences Center, Lubbock, Texas, United States; Texas Tech University, Lubbock, Texas, United States; Texas Tech University, Lubbock, Texas, United States

## Abstract

Caring for individuals with dementia presents a significant challenge, often placing caregivers under immense stress. Research has consistently highlighted that caregivers of individuals with dementia face an elevated risk of experiencing health concerns themselves due to the high stress and burden. This burden extends beyond psychological strain, with studies indicating higher rates of neurocognitive decline and pre-death grief (PDG) among family caregivers. Despite growing recognition of these health risks, there is currently a lack of interventions that target the family caregivers of persons with dementia. This study implemented 12-weeks of 3-hours of respite. We assessed overall neurocognitive functioning and PDG in caregivers at baseline and follow-up. Pilot data from 8 caregivers were analyzed. Assessments comprised of the Repeatable Battery for the Assessment of Neuropsychological Status (RBANS; i.e., attention, language, visuospatial ability, and immediate/delayed recall) and the PG-12 (i.e., pre-death grief). The individual reliable change index (RCI) for each participant was calculated as the difference in overall scores on the RBANS and PG-12 scores at baseline and follow up. Average PG-12 scores decreased (RCI = -0.88) at intervention, indicating a decrease in PDG. Further, RBANS (RCI = 1.2), immediate memory index (RCI = 4.58), language index (RCI = 3.16), attention index (RCI = 1.58), and delayed memory index (RCI = 2.33) scores increased. However, average visuospatial/constructional index (RCI = -8.04) scores decreased. These preliminary results suggest that only 3 hours of respite a week has the potential to reduce PDG and improve neurocognitive functioning among family caregivers of persons with dementia.

